# Perceived ethical acceptability of financial incentives to improve diabetic eye screening attendance

**DOI:** 10.1136/bmjdrc-2015-000118

**Published:** 2015-11-26

**Authors:** Hester Wadge, Colin Bicknell, Ivo Vlaev

**Affiliations:** Division of Surgery, Department of Surgery and Cancer, St Mary's Hospital, London, UK

**Keywords:** Behavior Modification, Ethics, Screening Strategies, Health Psychology

## Abstract

**Objective:**

To test the ethical acceptability of using financial incentives to increase diabetic retinopathy screening attendance.

**Background:**

Financial incentives could be an effective way to increase attendance at screening for diabetic retinopathy, although there can be ethical concerns about this approach.

**Design:**

Survey of people with diabetes in North West London. Those who were due to attend a screening appointment were invited to complete a questionnaire. Key demographic variables included age, gender, and deprivation.

**Setting and participants:**

A questionnaire was issued to those invited to attend screening in North West London and those who run the screening service. The questionnaire captured views on aspects of the ethical problem and different incentive types.

**Main variables studied:**

It captured views on the different dimensions of the ethical problem and different types of incentive. In order to understand how views might vary within a population, demographic variables were used to analyze the results.

**Results and conclusions:**

Vouchers were found to be the most acceptable form of incentive, significantly more so than cash payments. Most rejected the notion of targeting those who need incentivizing, preferring equality. Age was an important factor, with those aged between 40 and 64 the most optimistic about the potential benefits. Higher levels of deprivation were linked to increased acceptability scores. While some ethical concerns are strongly held among certain groups, there is also much support for the principle of incentivizing positive behaviors. This paves the way for future research into the effectiveness of incentivizing diabetic retinopathy screening attendance.

**Key messages**

- Financial incentives could reduce inequalities by improving health screening uptake; however, there are ethical concerns about the use of financial incentives in health.
- We asked people with diabetes about their views on the acceptability of incentives.
- Vouchers were more acceptable than cash payments, and those in deprived and middle-aged groups found incentives most acceptable.

## Introduction

Deprivation is linked to higher levels of diabetes,^1^ worse outcomes, and the development of secondary associated conditions. Retinopathy complications are the top cause of blindness in the UK working-aged population.^2^ In areas with the greatest socioeconomic deprivation, diabetes prevalence is highest and screening attendance is lowest.^3^ ^4^ Demographic factors such as age and deprivation are predictors of adherence to screening programs.^5^ If screening programs only reach the less deprived, they have the potential to exacerbate health inequalities.

Financial incentives have successfully changed organizational behavior, paying health providers more for improved performance.^6^ Incentives are also a powerful mechanism to encourage healthy behaviors such as smoking cessation.^7–9^ Research into whether financial incentives increase participation is largely confined to immunization initiatives.^10^ Compared with disincentives and increased regulation, incentives could be popular;^11^ however, ethical concerns around coercion, personal responsibility, and unintended consequences have inhibited research.^12^

This study explores these concerns with people who have diabetes and their health professionals. The primary objective was to determine whether these groups find the use of financial incentives in screening ethically acceptable. We analyzed the impact of demographic profile and different incentives on perceived acceptability.

Acceptability is a difficult term—this study did not attempt to interrogate moral acceptability, which tends to be inflexible and abstract. Nor was it primarily concerned with personal acceptability, which can be selfish. The ethical acceptability here refers to societal norms, which can be flexible and reflect personal experience and demographic group.

## Methods

We used a questionnaire methodology, as this allowed for the collection of comparable data from a broad sample. Questionnaires also allowed for privacy and anonymity, and minimal participant time commitment.

### Participants

The study was conducted across west London, in partnership with local provider 1st Retinal Screen Ltd. All 925 people with diabetes due to be screened in August 2013 were approached. Of these, 828 attended their appointment, during which they were issued with a voluntary questionnaire. There was a response rate of 44%. Questionnaires were posted to those who did not attend, with an invitation to rearrange their appointment. The views of this cohort would be especially valuable, as the success of an incentive scheme would be judged on an increase in participation; however, only six responded.

Finally, to ascertain the perspective of diabetes professionals, a questionnaire was issued to the screening team at 1st Retinal Screen, and to all general practitioner (GP) practices in the area for which addresses were available. The screening team were very engaged with the project and all five completed the questionnaire. None of the local GP practices responded.

### Questionnaire

For attendees and non-attendees, questions 1–3 captured basic demographic information: gender, age group, and postcode.

Question 4 comprised statement pairs expressing opposing positions on ethical concerns raised in previous studies.^12^ ^13^ Owing to the complex nature of ethical debate and the difficulties in coding this into statements, it was not always possible to devise pairs that were logical opposites. However, the intention was to encourage participants to decide in favor of or against incentives.

*4.1: The message*

- Offering an incentive sends a positive message to everyone that participation in the screening program is important.
- Offering an incentive sends out the wrong message—people should not be paid to do the right thing—they should participate in screening for the good of their own health, not for money.

The way health services are delivered can communicate underlying messages about health and the National Health Service (NHS). This question captured views on how that message might be interpreted by the target group.

*4.2: Fairness/responsibility*

- At the moment, only some people get screened. If this scheme encourages everyone to be screened, this is fairer for society and means everyone has the same chance to be healthy.
- This ignores where the real responsibility lies. Doctors, the Government, and the NHS should encourage people to look after themselves without paying.

Incentive schemes may reduce health inequalities, but this could be perceived as the NHS ignoring its other responsibilities.

*4.3: NHS values*

- Incentives complement the values of the NHS, by encouraging those most in need to access the resources that will improve their lives.
- Incentives undermine the key principles of the NHS, that healthcare should be free when using services.

Key NHS principles are equality of access and the absence of financial transaction. Financial incentives may reduce inequality, but introduce cash transfers at the point of care.

*4.4: Autonomy/choice*

- It is better than compulsory screening—people can still choose whether to take the incentive.
- Very poor people may feel that they do not have a choice—offering an incentive might coerce, or force people into doing something they do not want to do.

Another key debate around the remit of the NHS is on consent. If incentives are perceived to threaten autonomy, this could render them unacceptable.

*4.5: Wider impact*

- If it works, it could be used in other services as a way of encouraging people to look after their health.
- It could lead to people expecting money to use other NHS services, which could be expensive in the long run.

Introducing a new and effective mechanism in one service could lead to proliferation.

*4.6: Opportunity cost*

- Offering incentives will cost the NHS less in the long run by preventing bad health before it becomes a problem.
- Taxpayers’ money should not go to people who are not looking after themselves properly.

Should the NHS be a passive provider of care for those who seek it, or take extra steps to combat problems early on?

*4.7: Risk*

- Even if screening can never be 100% accurate, if it prevents one person from losing their sight, it is worthwhile.
- Incentives would only be acceptable if screening was 100% accurate, because people would trust the result.

This question attempts to capture the problem of inaccuracy and risk. The NHS makes a concerted effort to communicate the fallibility and stress of screening. Taking stronger measures to encourage participation acts against these warnings, and may persuade those who would otherwise decide against screening.

Question 5 asked participants to consider different types of incentive. A cash reward is often found to be least acceptable but most effective,^14^ so it would be worth measuring the difference in opinion between cash and healthy food or book vouchers (5.2 and 5.1, respectively) and between covering expenses and additional reward (5.6 and 5.7). These questions test the ethical problem with cash payments against encouraging positive behavior and compensation, respectively.

The principles of behavioral economics suggest that we overweight small probabilities, which might make a prize draw effective.^15^ Alternatively, a financial reward every time might be more acceptable (5.10 and 5.9).

For the individual, a large incentive (eg, £60) might be a more agreeable prospect. Equally this might be perceived as wasteful and a smaller reward (eg, £6) might be perceived as more proportionate (5.3 and 5.4).

Targeting certain demographics could help further reduce inequalities and make a scheme more affordable.^16^ However, in a health system underpinned by solidarity and equality, targeted rewards could cause resentment. We asked whether incentives should only be available for those who had not attended before, or for all (5.7 and 5.8).

For those who did not attend questions 6–8 ascertained their reasons, to identify whether they had reservations or were misinformed. A previous study into the reasons for non-attendance informed the question design.^5^

The questionnaire was tested on patients with diabetes as well as patients without diabetics.

### Analysis

The high response rate from the attendee group allowed full statistical analysis. However, the markedly low response rate from the non-attendees and health professionals only permitted limited analysis.

Postcodes gave an Index of Multiple Deprivation score, which allowed the group to be divided into deprivation quintiles, with 1 representing the least deprived and 5 representing the most deprived. Simple summary statistics demonstrated the difference in acceptability for question 4.

**Figure 1:**
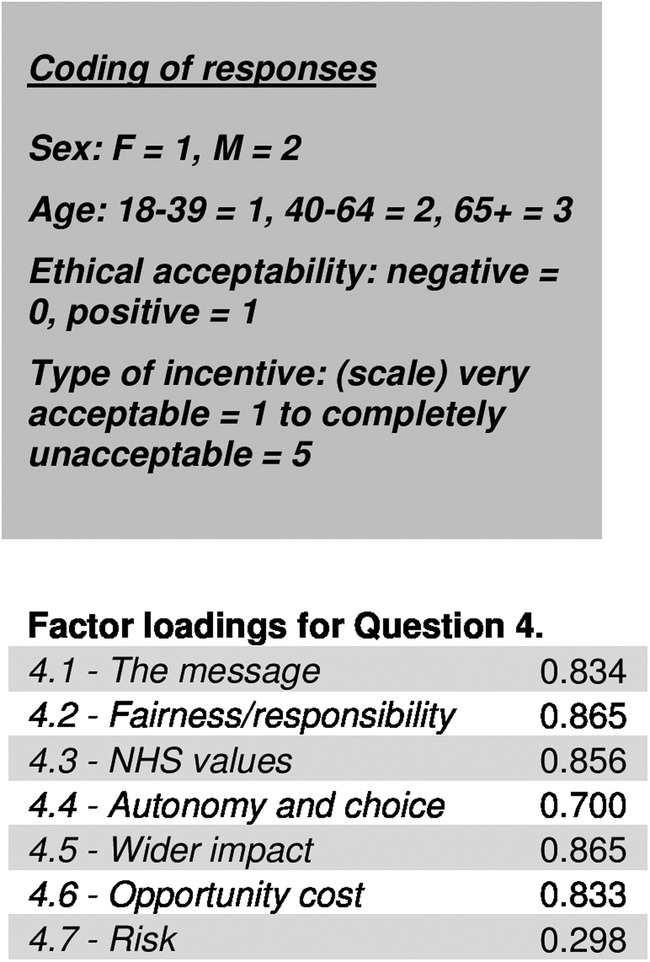
Factor analysis showing correlation between responses to question 4 (Cronbach's α coefficient measure of internal consistency). (NHS, National Health Service).

To ensure question 4 was capturing ethical acceptability, we ran a factor analysis which showed a high degree of correlation between responses (figure 1). The exception being the final statement pair. This outlier question may draw on different constructs, about the risks of screening itself, rather than how this relates to ethical acceptability. Cronbach's α coefficient measure of internal consistency found responses to question 4 to be highly reliable (α=0.91) when 4.7 was excluded.

By coding negative views as 0 and positive as 1, giving equal weight to questions 4.1–4.6, a mean score for participants’ perception of ethical acceptability was devised. Zero indicated that incentives were found to be unacceptable for all subquestions, and 1 indicated that incentives were found to be acceptable for all (table 1).

**Table 1:** Percentage of positive responses when investigating participants' perception of ethical acceptability

Question	Percentage of positive responses													
	Age									Deprivation quintiles				
	Female			Male			All			Least deprived			Most deprived	
	18–39	40–64	65+	18–39	40–64	65+	18–39	40–64	65+	1	2	3	4	5
4.1—The message	33.33	34.38	34.04	44.44	52.17	31.46	41.67	45.81	28.57	30.3	36.76	37.5	45.59	44.45
4.2—Fairness/responsibility	33.33	43.75	34.55	85.71	53.51	48.15	70	50	42.03	39.06	50.88	46.77	53.13	49.21
4.3—NHS values	33.33	43.55	32.69	50	47.37	37.04	45.45	46.02	34.81	34.38	44.46	39.34	50	44.45
4.4—Autonomy and choice	66.67	60.66	47.17	83.33	63.96	56.41	77.78	62.79	51.88	59.38	60	55.17	62.9	57.63
4.5—Wider impact	33.33	47.54	42.59	50	57.02	46.15	45.55	53.11	44.78	37.1	53.45	44.26	57.14	56.45
4.6—Opportunity cost	0	57.89	47.06	83.33	59.82	45	55.56	59.17	45.11	41.67	53.57	58.62	65.08	49.15
4.7—Risk	66.66	74.19	73.77	87.5	77.57	84.71	81.82	76.33	80.41	80.95	79.37	80	73.85	79.37
Overall acceptability (excluding 4.7) 0=completely unacceptable 1=very acceptable														
Mean	0.33	0.46	0.39	0.53	0.55	0.40	0.48	0.52	0.39	0.37	0.47	0.46	0.55	0.48
SD	0.44	0.41	0.40	0.46	0.42	0.40	0.44	0.42	0.40	0.40	0.41	0.42	0.41	0.43

NHS, National Health Service.

To understand the effect of sex, age, and deprivation on responses to question 4, a binary logistic regression was used, and a linear regression analysis on the overall mean acceptability score (table 2). A linear regression analysis on question 5 helped to understand the relative impact the demographic variables had on the perceived ethical acceptability for each type of incentive (table 3).

**Table 2:** Binary logistic and linear regression analyses to investigate the effect of sex, age, and deprivation on responses to question 4 using the overall mean acceptability score

Predictors	Binary logistic regression							Linear regression Overall acceptability (excluding 4.7)
	4.1	4.2	4.3	4.4	4.5	4.6	4.7	
	The message	Fairness/ responsibility	NHS values	Autonomy/ choice	Wider impact	Opportunity cost	Risk	
Sex								
β	0.511	0.481	0.133	0.197	0.294	0.021	0.356	0.052
p	0.036	0.045	0.587	0.423	0.223	0.932	0.205	0.266
OR	1.667	1.617	1.142	1.218	1.341	1.021	1.428	
Age								
β	−0.540	−0.349	−0.403	−0.460	−0.191	−0.412	0.258	−0.092
p	0.011	0.103	0.060	0.040	0.369	0.059	0.298	0.025
OR	0.583	0.705	0.668	0.631	0.827	0.662	1.295	
Deprivation								
β	0.138	0.083	0.088	−0.029	0.167	0.085	−0.028	0.026
p	0.097	0.313	0.284	0.736	0.044	0.311	0.778	0.105
OR	1.148	1.086	1.093	0.972	1.182	1.089	0.972	
R^2^	0.063	0.035	0.024	0.023	0.029	0.023	0.013	0.029

NHS, National Health Service.

**Table 3:** Linear regression analysis to investigate the effect of sex, age, and deprivation on responses to question 5 using the overall mean acceptability score

	Linear regression									
	5.1	5.2	5.3	5.4	5.5	5.6	5.7	5.8	5.9	5.10
Predictors	Vouchers	Cash	Small payment	Large payment	Expenses	Extra reward	Targeted	For all	Pay every time	Prize draw
Sex										
β	0.083	−0.204	−0.044	−0.075	−0.022	−0.295	−0.180	−0.091	−0.023	−0.098
p	0.663	0.284	0.822	0.662	0.910	0.088	0.344	0.660	0.904	0.599
Age										
β	0.405	0.298	0.257	0.420	−0.125	0.516	0.178	0.482	0.207	0.302
p	0.016	0.073	0.134	0.005	0.452	0.001	0.281	0.008	0.220	0.065
Deprivation										
β	−0.015	−0.106	−0.181	−0.100	0.030	−0.052	−0.037	−0.107	−0.098	−0.143
p	0.818	0.102	0.007	0.089	0.651	0.368	0.564	0.129	0.135	0.025
R^2^	0.020	0.027	0.035	0.042	0.003	0.056	0.009	0.035	0.014	0.033

β, regression coefficient; p, significance; NHS, National Health Service.

## Results

### Participants attending the clinic

Sixty-two percent of participants felt that offering an incentive sends out the wrong message and that people should participate in screening for their own good. Participants also showed concern about the way incentives align with the principles of the NHS, with 58.7% of those who answered feeling that it undermines the lack of financial transaction at the point of healthcare delivery.

Responses were balanced on whether it could be a useful tool elsewhere, or simply lead to people expecting payment in other services, with half (50.6%) fearing this possibility.

There was less concern about the potential for coercion and impact on the ability to choose, with 58.6% feeling that individual autonomy was retained. There was also slightly less concern about using health resources in this way, with more participants (53.1%) thinking it might cost the NHS less in the long run by preventing ill health. In total, 52.8% of participants felt that other routes should be tried, and more responsibility placed on the NHS to encourage people to take better care of themselves.

Overall, participants tended to answer consistently negatively or positively, with 31.3% answering negatively on all questions, and a further 11.2% erring on the side of unacceptability for all but one of the questions. In total, 27.7% responded positively to all questions with a further 5.6% answering positively to all but one.

Those in the middle age group were more accepting of incentives generally (mean=0.52, SD=0.42), which is especially evident for questions on the impact of autonomy (4.4) and on opportunity cost (4.6), which were answered positively by 62.79% and 59.17% of participants, respectively. In general, the older age group gave more negative responses, particularly on the message incentives send out, with only 28.57% giving a favorable view. Overall, participants in the two most deprived quintiles felt more positively about incentives than those in the least deprived groups (table 1).

Overall acceptability of incentives decreases with age in a statistically significant way (β=−0.092, p=0.025). Increasing age was also found to be a significant predictor on some of the ethical dimensions. Older participants were more likely to be concerned about the message (β=−0.54, p=0.011) and the impact on autonomy and choice (β=−0.46, p=0.04; table 2).

Deprivation and sex were not found to be significant predictors of overall acceptability; however, male participants were more likely to feel positively about the message (β=0.511, p=0.036) and issues of fairness and responsibility (β=0.481, p=0.045). Those in the most deprived groups were statistically more likely to feel positive about the wider impact of incentives (β=0.167, p=0.044; table 2).

On incentive types, vouchers were preferred to cash, with 36.5% of participants who answered the question finding vouchers to be very acceptable, compared with 18.6% who found cash very acceptable and 64.4% of participants finding it completely unacceptable. When asked whether small or large cash incentives would be preferable, most responded that neither would be acceptable. However, among those who responded positively, almost twice as many (65) found small incentives very acceptable than those finding large very acceptable (38).

Compensating for expenses incurred was found to be either slightly or completely acceptable by over half (51.1%) of all those who responded. This compares to 72% of participants who felt paying over and above reasonable expenses to be slightly or completely unacceptable. In total, 24.4% of participants found targeted incentives to be wholly or slightly acceptable, whereas 34.5% found incentives for all to be acceptable.

More deprived groups were statistically more likely to find small payments (β=−0.181, p=0.007) and prize draws acceptable (β=−0.143, p=0.025). With increasing age, vouchers were found to be more acceptable in a statistically significant way (β=0.405, p=0.016). Other statistically significant preferences for incentive types which increase with age were large payments (β=0.42, p=0.005), paying more than just expenses (β=0.516, p=0.001), and incentives for all (β=0.516, p=0.008; table 3).

The results demonstrate a clear preference overall for offering vouchers and expenses over cash and paying more than just expenses. The mean responses to question 5 also demonstrate a clear preference overall for vouchers compared with any other type of incentive. Small cash payments were, in general, preferred to large—although overall the mean score demonstrates less approval of cash payments in general, regardless of size. Prize draws were also found to be more unacceptable than small payments every time; however, this question was framed in the context of cash payments, and the mean responses reflect a general aversion to cash incentives (table 4).

**Table 4:** Mean scores for domains investigated in question 5 by age and deprivation quintile

		Mean and SD (scale: 1 (very acceptable)—5 (completely unacceptable))							
					Deprivation quintiles				
		Age groups			Least deprived			Most deprived	
Question		18–39	40–64	65+	1	2	3	4	5
Vouchers									
5.1	Mean	2.45	2.59	3.01	2.98	2.83	2.62	2.58	2.92
	SD	1.63	1.55	1.68	1.69	1.69	1.62	1.59	1.58
Cash									
5.2	Mean	4.33	3.69	4.21	4.16	4.14	3.96	3.43	4.00
	SD	1.23	1.70	1.45	1.42	1.49	1.66	1.82	1.56
Small payment (eg, £6)									
5.3	Mean	3.80	3.55	3.94	4.20	3.73	3.93	3.33	3.52
	SD	1.62	1.63	1.62	1.37	1.69	1.54	1.69	1.77
Large payment (eg, £60)									
5.4	Mean	4.33	3.96	4.58	4.51	4.40	4.31	3.73	4.28
	SD	1.37	1.59	1.12	1.15	1.29	1.35	1.71	1.48
Expenses									
5.5	Mean	3.09	2.66	2.57	2.68	2.38	2.80	2.37	2.91
	SD	1.64	1.56	1.65	1.61	1.53	1.67	1.37	1.79
Extra reward									
5.6	Mean	4.36	3.79	4.53	4.34	4.18	4.07	3.84	4.14
	SD	1.29	1.56	1.09	1.12	1.44	1.49	1.63	1.38
Targeted									
5.7	Mean	3.25	3.65	3.85	3.82	3.79	3.62	3.94	3.52
	SD	1.76	1.58	1.56	1.50	1.59	1.58	1.48	1.71
For all									
5.8	Mean	3.75	3.11	3.85	3.95	3.53	3.27	2.89	3.63
	SD	1.60	1.81	1.67	1.51	1.80	1.88	1.81	1.71
Payment every time (eg, £6)									
5.9	Mean	4.27	3.58	3.99	4.03	3.78	3.95	3.67	3.53
	SD	1.27	1.62	1.58	1.44	1.68	1.57	1.61	1.70
Prize draw (eg, £6000)									
5.10	Mean	4.55	3.65	4.24	4.16	4.28	4.11	3.40	3.87
	SD	.69	1.71	1.36	1.28	1.37	1.52	1.79	1.68

Using a paired samples t test to compare incentive types, vouchers were found to be significantly more acceptable than cash (t=−10.380, p=0.000). Small payments were more acceptable than large (t=−5.048, p=0.000). Expenses were significantly more acceptable than paying extra (t=−12.886, p=0.000).

### Non-attendees—postal responses

There were a range of views on ethical acceptability in general and for different incentive types, but mostly positive. While it is not possible to draw conclusions or properly analyze the non-attendee group, the mean acceptability for different incentive types broadly aligns to the responses provided by the attendee group, with participants preferring vouchers, small payments, expenses, and incentives for all.

### Staff

On overall ethical acceptability, there was a marked difference from the non-attendee group. There were far more negative responses than positive, with only 41% of all responses to question 4 falling in favor of incentives. On incentive types, staff gave responses broadly in line with those of both patient groups, demonstrating a preference for vouchers and expenses, on average finding these to be acceptable. While neither small nor large cash payments were found to be particularly acceptable, there was a clear preference for smaller incentives. The staff were the only group to prefer a prize draw over payment for all.

## Discussion

This study has shown that, across those surveyed, opinion is heavily polarized. Participants were more likely to find incentives ethically acceptable or unacceptable across all counts than to give a mixture of answers.

The ethical concerns expressed most strongly across the sample group were that incentives undermine the responsibility of Government and the NHS to promote healthy behaviors, but also of individuals to look after themselves. It was felt that financial transactions sat uncomfortably with the principles of the NHS. This suggests that the most pressing concern is about how financial incentives may derail existing cultural norms around responsibility. One interpretation could be that there is an ingrained societal understanding of the roles and responsibilities between patients, Government, and healthcare professionals, and uneasiness about how introducing financial transactions may affect this delicate balance. This should be seen in the context of the broad program of change from which the NHS has recently emerged, and may reflect a wider sense of anxiety about the state of the NHS, and uncertainty about how services will be delivered in the future.

Age was an important factor, with those in the middle age group most likely to find incentives acceptable. That this group has the most positive view should offer hope to proponents of incentives, as people currently in this age group are at the greatest risk of developing type 2 diabetes in the near future. Incentives may be a useful way to encourage early positive interaction with the necessary screening regime. Those above the age of 65 are much more likely to have a negative view of this approach. It is therefore vital for health organizations to have a full understanding of the age profile of their population in order to communicate and design a successful incentive scheme.

Across all groups, there was a strong preference for healthy food or book vouchers over cash, and only covering incurred expenses. This suggests that incentives can be framed in a more positive light if they are shown to fulfill an additional purpose, over and above encouraging greater participation.

For the patient groups, incentives which are targeted at those most in need of screening were found to be less acceptable than incentives for all. This supports the interpretation that there is less concern about escalating costs to the public purse, and suggests an aversion to perceived injustice. Equality, where all are given the same offer regardless of circumstance, may be a more important pillar of the NHS than equity, where resources are allocated according to need.

In considering how to implement any kind of financial incentive scheme, policymakers could look to the results of this study for insight. These results support previous studies which show that vouchers which can be used for healthy foods are often found to be more acceptable than cash.^13^ This may be because healthy food is seen as a treatment for wider health problems, helping make diabetes more manageable, rather than a reward for attendance. There is the opportunity here to use incentives to achieve other policy goals, such as healthy eating or physical activity through gym membership.

This study also points to the possibility that ethically acceptable ways of implementing incentive schemes could align well with another universal policy goal: achieving more with fewer resources. Participants in this study generally preferred small to large payments and favored an approach that solely reimbursed reasonable expenses—this is especially true for the most deprived. This group also found prize draws to be more acceptable, which would be more cost-effective than paying all attendees.

In revealing that those in the most deprived groups find incentives more acceptable than those in the least, this study suggests that financial incentives could be used as a lever to reduce inequalities. If universal incentive schemes can have targeted appeal, they could raise the health status of the most deprived in our communities.

If incentive schemes can be found to be acceptable and practicable for diabetic retinopathy screening, other national or local screening programs might find it a useful tool, for example, for breast, cervical and bowel cancer, and abdominal aortic aneurysm. Policymakers would be advised to proceed more carefully here, as some of these schemes are screening for arguably more serious conditions, whereas others may involve more invasive methods or subsequent treatment, such as a risky operation. This leads to a shift in the ethical dimensions for different types of screening.

### Limitations

As an initial investigation, this study has revealed some useful and important findings. An expanded study, with a more refined questionnaire aimed at reaching a wider and more diverse sample group would allow inferences to be made about the whole population. A sample which assessed the views of those from different areas of the country would have been useful; as urban Londoners are not necessarily representative of the country as a whole. The sample was also largely comprised of those who might benefit from an incentive scheme. There is a concern that responses may be distorted in self-interest.

We experienced difficulty in gathering the views of those who did not attend their screening appointment: while this was anticipated, it is nonetheless frustrating, as this is the group that an incentive scheme would ultimately target. It might have been more productive to call each non-attendee directly and guide them through the questionnaire over the telephone.

In asking participants to choose from predetermined ethical concerns, the argument was framed, potentially influencing participants’ views. A more open approach which asked participants for their views on incentives with fewer prompts may have been less subject to potential bias in questionnaire design.

The number of people who chose to make additional comments suggests that a free-text field in the questionnaire would have been welcomed and allowed the collection of qualitative data. Some participants expressed difficulty as the ethical statement pairs were not mutually exclusive, and that this made it difficult to choose. Others felt they appreciated both sides of the argument. Further research into this subject might want to allow participants more time for consideration, and revisit the questionnaire design to provide more detailed or granular questions.

There is certainly space for further qualitative, quantitative, and philosophical research into the ethical acceptability of incentives, and their implementation and efficacy.

## Conclusion

This preliminary study suggests that some groups may find financial incentives ethical acceptable, although there will be many who take the opposite view. Replication and extension of this study may offer further insight, and research into the efficacy of incentive schemes in increasing screening program participation will be essential to the development of our understanding in this area.
